# Books and Authors

**Published:** 1911-04

**Authors:** 


### BOOKS AND AUTHORS

**Systemic (including Special) Pathology.** By **J. George Adami,** M.A., M.D., LL.D., F.R.S., Professor of Pathology, and **Albert G. Nicholls,** M.A., M.D., F.R.S., Assistant Professor of Pathology in McGill University, Montreal. In one octavo volume of 1,160 pages with 301 engravings and 15 plates. Philadelphia and New York: Lea & Febiger. 1911. (Cloth, $6.00, net.)

This single volume covers the whole subject of special pathology, and it would have been considerably smaller if it were not distinguished from other works by two very valuable features; the inclusion of the blood and circulatory organs and the treatment accorded to disturbances of function as well as of structure. Obviously, function and its derangements are what the clinician and practitioner most wish to understand, and structure is merely a step thereto. As a connecting link, therefore, between theory and practice, functional pathology is of prime importance, and a work properly emphasising it is indispensable alike to the student and practitioner. Indeed, the keynote of the book is to link theory to its practical application, and to be suggestive and helpful.

Among the changes in this edition is that the chapters on the ductless glands and the nervous system have been rewritten, the latter almost recreated. The section on the urinary system has been greatly amplified and the chapters on the circulatory and respiratory systems have been made more logical, coherent and adequate than before. In the new edition, Adami has written practically the whole of the introductions to the circulatory and respiratory systems and the ductless glands and about half of that on the urinary function, the remainder of the work being from the pen of Dr. Nicholls. The second edition will increase the favor with which the original was received.

---

**Bismuth Paste in Chronic Suppurations**. By **Emil G. Beck,** M.D., Surgeon to the North Chicago Hospital. With an Introduction by **Carl Beck,** M.D. Octavo, pp. 237. Illustrated. St. Louis: C. V. Mosby Co. 1910. (Cloth, $2.50.)

In this monograph the author gives the results of his experience with the use of bismuth paste in the treatment of certain chronic suppurative lesions, notably bone and other sinuses. After a brief introduction and consideration of general conditions a chapter is devoted to bismuth paste in anatomical diagnosis, followed by one on diagnostic errors revealed by injections of the paste. In the chapters following the technic employed is fully described and many interesting cases are reported showing the efficacy of bismuth paste in the treatment of fistula, sinuses, pus cavities and other conditions. The section on bismuth poisoning and its prevention adds to the practical value of the book. Joseph C. Beck contributes a chapter on bismuth paste in the treatment of chronic suppurative diseases of the nose, accessory sinuses, ears and mastoid processes, while the closing chapter summarises the experiences of Rudolph Beck and of other dentists in the management of pyorrhea alveolaris with bismuth paste.

Numerous radiographic illustrations through the book give emphasis and clearness to the text. It is a work of art mechanically speaking, reflecting credit alike upon the author, artist, and publisher.

---

**Induced Cell-Reproduction and Cancer.** By **Hugh Campbell Ross,** M.R.C.S. (Eng.), L.R.C.P. (Lond.), Director of Special Researches at Royal Southern Hospital, Liverpool. Octavo, pp. 453. Illustrated. Philadelphia: P. Blakiston's Son & Co. 1911. (Cloth, $4.50.)

The most remarkable thing about this book is the enthusiasm and idealistic tendencies of the author who in a rather long and somewhat interesting preface tells a delightful story of how he happened to discover the in-vitro method of staining. It seems he was carrying on experiments with blood on nutrient agar at a temperature of 37 c in his laboratory on board a war ship. A midshipman set off a 12-inch gun and the concussion so shattered the incubator that it disarranged matters to an extent which bid fair to spoil the experiments under way. Examination later showed that the temperature had been sent up to 60 c and that the blood was diffused through the jelly. That is about the end result except that the author took advantage of the accident to work out his theory to the point where it became a book saleable to the profession.

There is little in the book that throws light on the cancer question and it remains just where it was before the gun shattered the incubator. Gaylord and Clowes of the Buffalo State Cancer laboratory are given credit for the investigations they are carrying out.

---

**Blakiston's Quiz Compend Series... Gynecology.** By **William Hughes Wells,** M.D., Associate in Obstetrics in the Jefferson Medical College. Fourth edition. 12 mo., pp. 290. Illustrated. Philadelphia: P. Blakiston's Son & Co. 1911. (Cloth, $1.00 net.)

Improvement is shown in the fourth edition of this practical compend on gynecology. All the obsolete operations and methods of technic have been eliminated. Everything that is new in the field of gynecology has been incorporated and all the important operations and new instruments are illustrated freely.

---

**Memoranda on Poisons.** By **Thomas H. Tanner,** M.D. Eleventh revised edition by **Henry Leffmann,** M.D. Philadelphia: P. Blakiston's Son & Co. (Cloth, 75 cents.)

Here is a little book, almost of vest pocket edition size which tells all about poisons and is as handy a little manual as has been issued. The fact that it has reached the eleventh edition speaks for its popularity. It has been brought up to date and contains notes on synthetic preparations used in place of morphine; the toxicology of poisonous foods and a revised chemical nomenclature. It is valuable from every viewpoint.

---

**Diagnosis and Treatment of Diseases of Women.** By **Harry Sturgeon Crossen,** M.D., Professor of Clinical Gynecology, Washington University, St. Louis. Second edition. Octavo, pp. 1025, with 744 engravings. St. Louis: C. V. Mosby Co. 1910. (Cloth, $6.00.)

Confined wholly to diagnosis and treatment of gynecologic conditions the first edition of this book met immediate favor and a second edition has now become necessary not because the first, owing to advances in gynecology became out of date, but because the edition was exhausted; because the demand for a sensible, common sense and intelligible book on women's diseases, all of which this work is, required a new supply. Taking advantage of this necessity Crossen has amplified his book and made of it as nearly a complete work as is possible. To the material contained in the first edition has been added two hundred pages of text and fifty original illustrations. A decided improvement, as those of us who have much to do with text books will appreciate, is the rearrangement of the index which has been brought to the highest state of efficiency. There are now references and cross references to every diagnostic mention in the book.

Pelvic inflammations and tubal pregnancies have been given special attention and these two items alone will amply repay the general practitioner for even a cursory reading of them. They will show him clearly and without any of those by-path musings habitual with many writers on medical matters, the correct route to definite diagnosis. There has been so much written and spoken concerning these conditions that is chaotic and impossible of approach, much less of acceptance, that directions of such clarity as distinguishes Crossen's writings are invaluable. Functional disturbances have received extended mention and careful consideration as regards diagnosis and treatment. A valuable addition to this edition is the introduction of medicolegal conditions. True, the space given this very important department of gynecology is limited but it opens the way for a more extended description of medicolegal conditions in subsequent editions.

The book is excellently written and may be accepted as one of the most useful of the gynecologies available.

---

**International Clinics.** A Quarterly of Illustrated Clinical Lectures and especially prepared articles on Treatment, Medicine, Surgery, Neurology, Pediatrics, Obstetrics, Gynecology, Orthopedics, Pathology, Dermatology, Ophthalmology, Otology, Rhinology, Laryngology, Hygiene etc. Edited by **Henry W. Cattell,** M.D. Vol. IV, twentieth series. Philadelphia and London: J. B. Lippincott Co. 1910. (Cloth, $2.00.)

There is special interest in this volume because of the description and references to Ehrlich's salvarsan and its use. Such marked advance has been recently made in the use of this drug that the article which leads in this volume may be considered as a basic writing. It is of extreme interest and is followed by reports of the use of "606" in syphilitic iritis.

Two articles of especial interest are "Physicians' Fees Down the Ages," by James J. Walsh, M.D., and "Wounds by Firearms," by Williams S. Wadsworth, M.D., which appear in the post-graduate course department. The former article is as complete as careful research can make it and as stated by the author it shows that from a period 4,000 B. C., up to the present the fees of physicians and surgeons have been based on the value of the benefit conferred on the patient—and the patient's ability to pay. Dr. Wadsworth's article on firearm wounds is exhaustive and medicolegally valuable.

**Mental Symptoms of Brain Disease.** By **Bernard Hollander,** M.D. With preface by **Dr. Jul. Morel,** late Belgian State Commissioner in Lunacy. 12 mo, pp. 255. New York: Rebman Co. (Cloth, $2.00.)

There is very much that is worth while in this book. The author places before us a great mass of material the result of his own experience and of his careful investigation in literature. We know of no other work which presents so many valuable facts in so small a compass.

He has shown beyond a reasonable doubt that certain cases of insanity have been due to local brain disease of limited extent. He has proven his thesis that some of these cases may be cured by operation.

He considers successively the mental symptoms in lesions of the frontal lobe, the parietal lobes, the temporal and the occipital lobes. We agree with Hollander's conclusion "In every case of insanity in which there is a history of head injury and the locality injured corresponds with the locality indicated by the symptoms, as described in this work, operation should positively be undertaken, even if there be no external sign of trauma." We take pleasure in recommending this work as a valuable aid to alienists.

---

**Principles of Therapeutics.** By **A. Manquat,** National Correspondent to the Academy of Medicine. Translated by **M. Simbad Gabriel,** M.D. Octavo, pp. 298. New York and London: D. Appleton & Co. 1910. (Cloth, $3.00.)

Therapeutics as a subject is not studied with the attention it deserves nor is it practised with sufficient precision. This book by Manquat and translated by Gabriel takes up and treats in detail "The Principles of Therapeutics" and deals with individual drugs scarcely at all.

The author begins Chapter VII with the true statement that the good physician is he who succeeds best in individualising his therapeutic schemes; that is in prescribing for a given patient the corrective which is exactly necessary for him. In subsequent chapters the relation of the physician to the patient, the relation of the attending physician to the consultant, the relation and duties of the nurse to both physician and patient is discussed in a readable and interesting manner. Several chapters are given over to a consideration of non-therapeutic agents.

---

**Handbook for Treatment of Diseases of the Eye.** By **Dr. Curt Adam,** Berlin. Translated by **William George Sym, M.D.,** and **E. M. Lithgow,** M.B. 12 mo, pp. 276. Illustrated. New York: Rebman Co. (Cloth, $2.50.)

Every physician in general practice should know, and usually does know how to treat the most common eye diseases that occur in his practice. It is the family doctor who is most frequently consulted,and called upon to deal with the ordinary eye troubles of his patients. This being so, it is important that he keep himself informed of the latest thought in this branch of medicine. Adam's little book is one of the best on the subject. It contains a vast amount of practical information stated in a brief and lucid manner. From the fact that a second edition has been called for within a year stamps it, and deservedly so, as a very popular manual.

